# Q Fever: An Emerging Reality in Portugal

**DOI:** 10.7759/cureus.19018

**Published:** 2021-10-25

**Authors:** Rita Lencastre Monteiro, Rui Nascimento, Joana Diogo, Rita Bernardino, Rodrigo Nazário Leão

**Affiliations:** 1 Internal Medicine, Hospital Santo António dos Capuchos - Centro Hospitalar Universitário Lisboa Central, Lisboa, PRT; 2 Radiology, Centro Hospitalar Universitário do Porto, Porto, PRT

**Keywords:** portugal, endemic, atypical pneumonia, coxiella burnetii, q fever

## Abstract

Q fever is a zoonosis caused by *Coxiella burnetii *with worldwide distribution at the increasing expression in Europe and endemic in Portugal. It is transmitted by inhalation of aerosols containing spores, main reservoir being cattle, goats and sheep as by ingesting cottage cheese or unpasteurized milk. The majority of patients are asymptomatic; however, they may present with fever, atypical pneumonia, acute hepatitis, cutaneous manifestations and rarely with cardiac or neurological involvement. Although most cases are self-limited, focal persistent or chronic Q fever can manifest years after the onset, wherefore follow-up is essential. The clinical heterogeneity may be so variable that the disease is often diagnosed only if it has been systematically considered. It should be especially taken into account in the presence of risk factors as valvular or joint prostheses, immunocompromised patients, pregnant women and epidemiological setting. The authors present a rare case of *Coxiella burnetii* pneumonia with cutaneous and hepatic manifestations without any risk factor. This case aims to emphasize the importance of Q fever in the differential diagnosis of fever or atypical pneumonia, even in the absence of known risk factors. The diagnosis is often challenging for clinicians and it is necessary to maintain a high index of suspicion. In Europe and specifically in Portugal is mandatory to report the cases to establish the real impact of this disease.

## Introduction

In Portugal although undiagnosed and underreported [1] it's a mandatory declaration diseases and according to the National Information System for Epidemiological Surveillance (SINAVE) with 87 reported cases between 2013 and 2016 [2]. Is considered an endemic disease with an incidence of 0.11 cases per 105 inhabitants and the highest number of reported cases in the Center and South regions [3,4]. In a case series of Q fever in Portugal 8% of the patients had no risk factors which may lead to diagnostic delay [1].

Q fever is transmitted by inhalation of aerosols containing spores present in infected animals with the main reservoir being cattle, goats and sheep. Infection can also occur by ingesting cottage cheese or unpasteurized milk. The disease has a predominance in adult men in active work between 30 and 60 years old [5].

The incubation period is two to three weeks and 60% are asymptomatically with only 2% needing hospitalization [6]. The primary infection can manifest in any organ and most cases are self-limiting, lasting one to three weeks, usually with a good prognosis [7]. The most frequent clinical manifestation of acute Q fever is a self-limited febrile illness, atypical pneumonia or acute hepatitis [6]. Cutaneous manifestations as maculopapular or vesicular exanthema and sometimes purpuric lesions occur in 1-9% [8]. More rarely, in 1% of the cases there is cardiac involvement as pericarditis, myocarditis or acute endocarditis and in less than 1% neurological implication as encephalitis or meningitis [4,5].

Focal persistent or chronic Q fever occurs less than 5% and can manifest months or years after the initial infection, mainly with localized clinical signs as endocarditis, chronic hepatitis, chronic vascular infections, osteomyelitis, osteoarthritis and chronic pulmonary infections [7]. Although patients likely have lifelong immunity to reinfection, disease recrudescence might occur and follow-up is essential for timely detection since chronic Q fever leads to morbidity and mortality rates up to 60% [9]. 

## Case presentation

A 59-year-old man with a medical history of essential hypertension begins complaining of fever with an axillary temperature of 39°C, dyspnea, chills, headache and nausea without cough, sputum or chest pain. No other focal complaints. He denied relevant epidemiological context. Respiratory infection was assumed and empirical antibiotics were started with amoxicillin 875mg + clavulanic acid 125mg bid associated with azithromycin 500mg id.

After 72 hours without improvement, the patient was referred to the emergency department. Clinically he was febrile at 39.4ºC with a maculopapular, non-pruritic, symmetrical exanthem in the axillary region with extension to the inguinal region and hemodynamically stable, eupneic with normal pulmonary auscultation.

**Figure 1:**
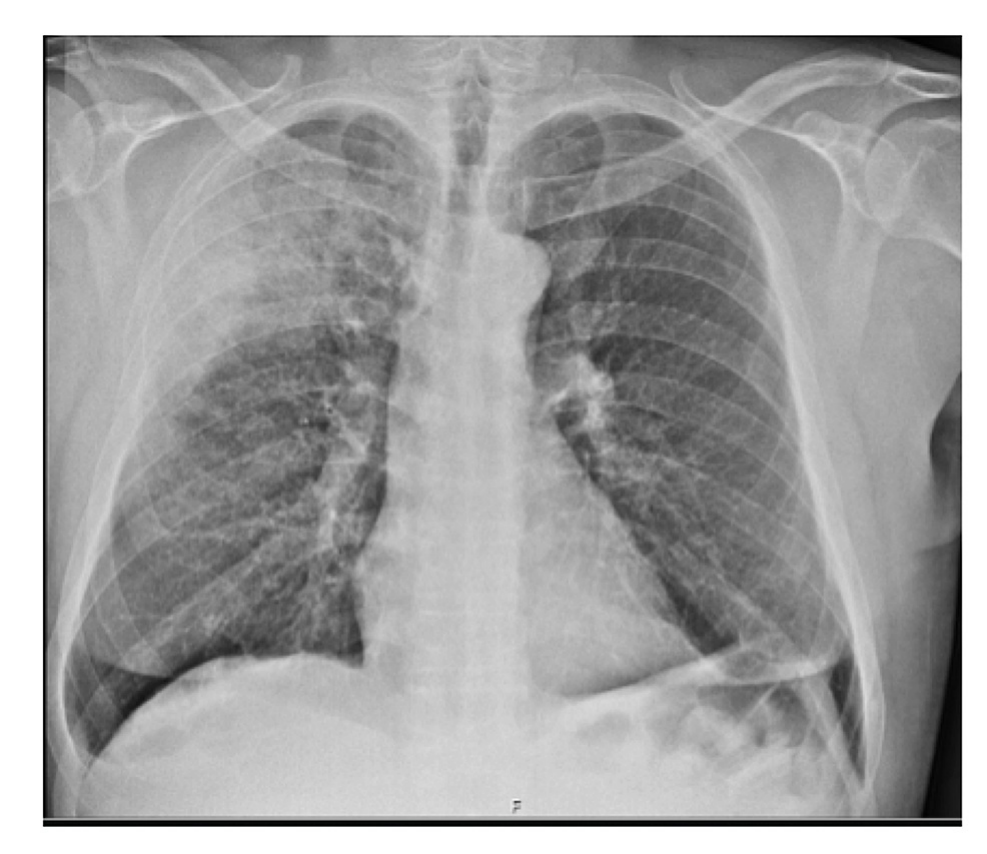
Anteroposterior chest radiography.

**Figure 2:**
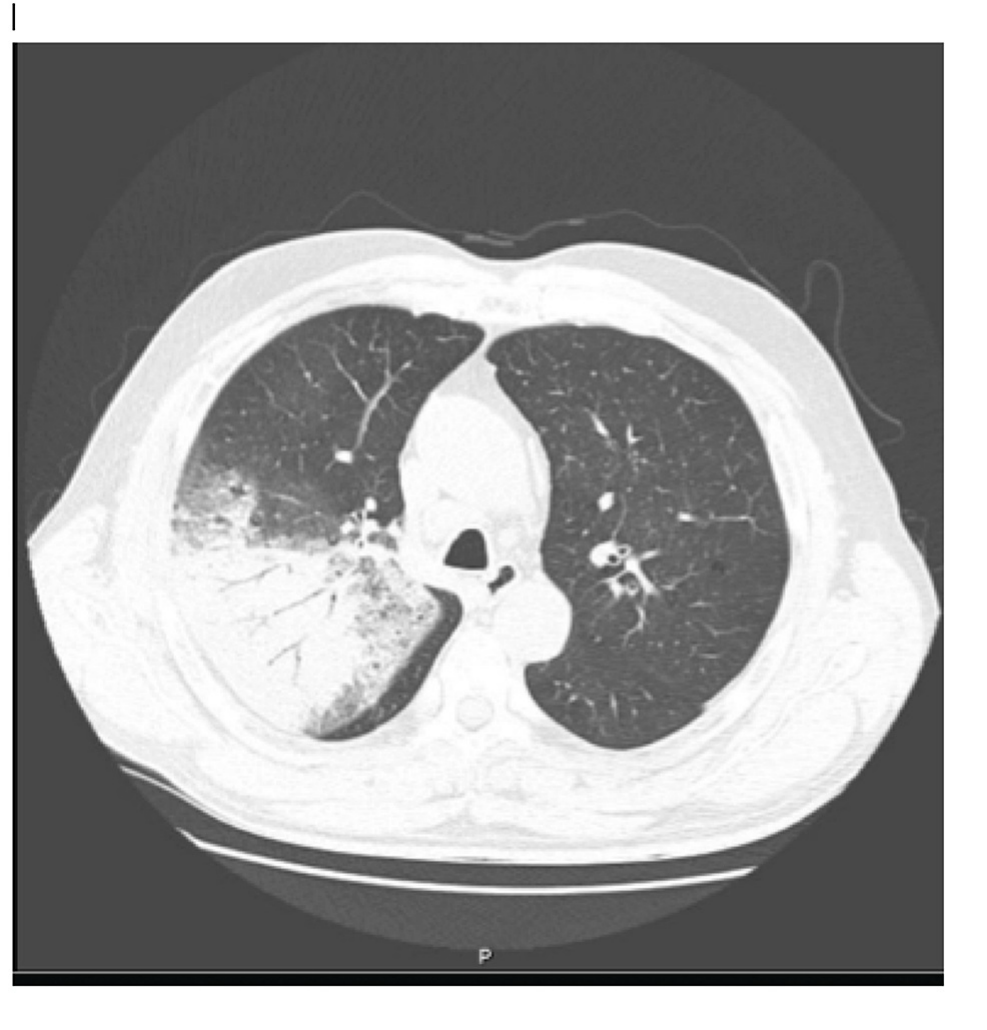
Thoracic computed tomography in the upper apex.

Lab tests shown no leukocytosis, C-reactive protein 324.5 mg/dL and increased cytocholestasis parameters revealed by aspartate aminotransferase 81 U/L, alanine aminotransferase 71 U/L, gamma-glutamyl transferase 106 U/L and alkaline phosphatase 206 U/L. Chest radiography (Figure 1) and chest computed tomography (Figure 2) reveal the presence of consolidation in the right upper lobe with air bronchogram.

The patient was admitted with atypical lobar pneumonia and started on empiric piperacillin/tazobactam 4.5g 4id for seven days. The extensive differential diagnosis that was considered is summarized in Table 1. At bronchofibroscopy the direct examination of bronchoalveolar lavage (LBA) revealed no microorganisms, including alcohol-resistant bacilli and the cultural examination was negative. LBA was positive for *Coxiella burnetii *and quantitative serologies had positive Immunoglobulins (Ig)M antibodies phase I and II and IgG antibodies positive for phase II and negative for phase I. Transesophageal echocardiography ruled out endocarditis. Given the benign course, previous antibiotic therapy, time of disease and exclusion of endocarditis no doxycycline was started.

**Table 1:** Differential diagnosis. AB: antibodies.

Etiologic study	Result (title)
Blood cultures	Negative
Influenza virus panel	Negative
Legionella pneumophila and Streptococcus pneumoniae antigenuries	Negative
Human Immunodeficiency Virus (HIV)	Negative
Hepatitis C virus (HCV)	Negative
Hepatitis B virus (HBV)	Negative
Rapid Plasma Reagin (RPR)	Negative
Mycoplasma pneumoniae IgG and IgM Antibodies	Negative
Chlamydia pneumoniae IgG and IgM Antibodies	Negative
Interferon-gamma release assay (IGRA)	Negative
Pneumocystis jirovecci Polymerase chain reaction (PCR) assay of bronchoalveolar lavage (BAL)	Negative
Chlamydia pneumoniae PCR assay of BAL	Negative
Coxiella burnetii PCR assay of BAL	Positive
AB Anti-Coxiella burnetii IgG (Phase I)	Negative
AB Anti-Coxiella burnetii IgM (Phase I)	Positive (1/50)
AB Anti-Coxiella burnetii IgG (Phase II)	Positive (1/200)
AB Anti-Coxiella burnetii IgM (Phase II)	Positive (1/100)

On re-evaluation at three and six months, the patient was asymptomatic with serological IgG antibodies phase I <200, so there was considered to be no progression to persistent infection or post-fever fatigue syndrome.

## Discussion

The clinical and imaging features of *Coxiella burnetii *are hardly distinguishable from other atypical pneumonias. The most characteristic signs are fever (82%-100%), headache (35%-100%), non-productive cough (60%-70%) chills (40%-88%) and chest pain (50%-60%) [10]. In the case presented here, the clinical presentation with fever, mild respiratory complaints, and cutaneous manifestations is noteworthy. Although classically Q fever is not often associated with skin lesions, a large epidemiological study from France showed that 20% of patients with acute Q fever presented with maculopapular or purpuric skin eruptions [11]. In the case presented here, no epidemiological context or associated risk factors were found, but these should be actively investigated. It is important to systematically question occupation, recent time spent in the countryside, contact with animals or consumption of unpasteurized dairy products [1]. There are also geographical variations in the clinic and in Portugal it manifests more often as an isolated febrile syndrome or with hepatic focalization [9]. 

In the matter of lab tests, the main features are the absence of leukocytosis (75%) and an increase in liver enzymes (85%) [7]. Microscopic haematuria is present in ∼50% of patients with Q fever pneumonia. Antimitochondrial antibodies, anticardiolipin antibodies and antismooth muscle antibodies have been described. On imaging exams, there is nothing distinctive [11]. In most instances the laboratory diagnosis is serological and the indirect immunofluorescence is the reference technique according to World Health Organization. Quantitative serologic testing with evaluation of IgG titers or detection of *Coxiella burnetii *by protein chain reaction (PCR) is recommended [3-4]. A four-fold rise in antibody between acute and convalescent samples is diagnostic with phase II immunoglobulin IgM titre of >1:64 or a phase II IgG titre of >1:256 is strong evidence of recent infection [12]. We can see from the case that the laboratory and imaging features are nonspecific and quantitative serological tests are crucial for the diagnosis. 

The therapy of choice for the acute disease is a two-week course of doxycycline because it reduces the duration of disease, symptomatology, and the risk of complications. Although tetracyclines, quinolones, rifampin and newer macrolides may have some beneficial effects due to its ability to permeable into the cells. The use of beta-lactams is more controversial since Coxiella burnetii is an obligate intracellular parasite and beta-lactam antibiotics barely permeate into the cells, however, there are many cases of spontaneous cure and it is likely that beta-lactam treatment may have been involved [13]. Alternative treatments with trimethoprim-sulfamethoxazole are recommended for pregnant women and young children [14]. Antibiotic combinations administered over prolonged periods are necessary to prevent relapses in Q fever endocarditis patients or if there is a high risk for persistent forms requires 18-24 months of doxycycline in combination with hydroxychloroquine. Although the protective role of Q fever vaccination with whole-cell extracts has been established, the population which should be primarily vaccinated remains to be clearly identified [10]. Treatment for acute Q fever is not routinely recommended for asymptomatic persons or for those whose symptoms have resolved, although it might be considered in those at high risk for developing chronic Q fever [7]. In the present case, the decision not to start doxycycline despite the isolation was due to the clinical improvement after 10 days of antibiotic therapy with beta-lactam and macrolide, as well as the absence of prostheses and the exclusion of endocarditis through echocardiography.

Immunocompromised patients are at higher risk of developing complications that can result in fatal interstitial pneumonia, myopericarditis or encephalitis. The infection of the vascular prosthesis and prosthetic valve infections can result in chronic infection. Chronic persistence present in 20% of cases can result in Q fever fatigue syndrome, hemolytic anemia and bone marrow necrosis [15]. In the presented case the patient had a good evolution confirmed by serological tests. 

## Conclusions

This article's purpose to grow awareness of this disease and alert to this entity, oriented towards a correct identification of the pathology. The case presented aims to emphasize the importance of Q fever in the differential diagnosis of fever encompassing a great clinical heterogeneity, which must be recognized even in the absence of an epidemiological context or in the absence of known risk factors as presented. That proves that it is necessary to maintain a high index of suspicion in clinical practice. Although Q fever is very prevalent in Europe as in Portugal it is frequently ignored and undiagnosed. Diagnosis is often missed due to mild symptoms and if is only made in a chronic phase with possible late consequences. It is very important to know the disease well in order to arise as a hypothesis and If confirmed it is mandatory to notify Public Health authorities and treatment can be initiated as soon as possible. It is of crucial to take more care in reporting within clinical practice to have grown data and knowledge about the real impact of this treatable and potentially serious infection.
